# How Will the Mild Encephalitis Hypothesis of Schizophrenia Influence Stigmatization?

**DOI:** 10.3389/fpsyt.2017.00067

**Published:** 2017-05-03

**Authors:** Sabine Müller, Rita Riedmüller

**Affiliations:** ^1^Mind and Brain Research, Psychiatry and Psychotherapy, CCM, Charité - Universitätsmedizin Berlin, Berlin, Germany

**Keywords:** schizophrenia, ethics, mild encephalitis, stigmatization, discrimination

## Introduction

People diagnosed with mental disorders, particularly those with schizophrenia, are severely stigmatized (1, 2). The image of people with mental disorders is strongly influenced by the mass media, which are then influenced by the prevailing medical opinion as well as by current research results. Therefore, researchers in psychiatry bear a certain responsibility for the stigmatization of their very own research objects.

Within the recent years, the mild encephalitis hypothesis receives more and more scientific interest. According to this hypothesis, a mild, but chronic, encephalitis underlies the symptoms of schizophrenia in a subgroup of patients. Infections, traumas, or autoimmune diseases can cause a mild encephalitis, which leads to psychiatric and/or neurological symptoms (3–5).

Since the mass media have recently started to report about the association of brain inflammation and schizophrenia, the mild encephalitis hypothesis is starting to influence the public’s opinion about people diagnosed with schizophrenia, and thus will have a certain influence on the stigmatization. Whether it will increase or decrease stigmatization has not yet been investigated empirically. In the following, we discuss this question on grounds of theoretical concepts and empirical research on stigmatization of schizophrenia.

## Stigmatization of Mental Disorders

Stigmatization is sociologically defined as the classification and stereotyping of people because of a negatively connoted attribute, together with segregation and loss of social status, discrimination in important contexts, and devaluation in a social hierarchy in a situation of exercise of power (6). Many stigmatized individuals internalize the negative evaluation, try to hide the negatively connoted attribute, and withdraw from society (self-stigmatization). Stigmatization often affects the social circle, particularly the families (courtesy stigma) (7).

Many biologically orientated researchers are convinced that biological explanations of psychiatric disorders will reduce stigma. This optimistic view is based on the attribution theory, assuming that the main reason for stigmatization is the attribution of guilt or responsibility for the onset and/or maintenance of the deviant behavior (8). Accordingly, biological, and particularly genetic, explanations should reduce blame against persons with mental disorders as soon as people understand that the strange or frightening behavior is not caused by evilness or weak will, but by a disease (9).

This conviction is contested by many social scientists. Because both the moral and the medical concepts assume an inborn predisposition for deviant behavior, a genetic explanation of deviant behavior does not diminish rejection (10). Genetic explanations assume mental disorders to be unchangeable, more serious, and hereditable (9, 11). People convinced of “genetic essentialism” believe that the genes are a person’s essence and that the characteristics and behaviors of a person are based on his/her genetic makeup (11). Genetic explanations increase self-stigmatization (12) and courtesy stigma, particularly the stigmatization of genetic relatives of people with mental illness (9). Furthermore, this approach supports a paternalistic attitude towards mentally ill persons, questioning their autonomy and decisional capacity (13).

The attribution theory and the concept of genetic essentialism are not mutually exclusive; rather they grasp different aspects of stigmatization: the first one mainly the attribution of guilt and the second mainly the fear and the feeling of social distance (10).

## Empirical Research on Stigmatization of Mental Disorders

Empirical research supports the theory of genetic essentialism and widely disproves the attribution theory for major depression and schizophrenia. For example, a representative study with 1,241 participants (9) confirmed only one prediction of the attribution theory, namely, that people who are convinced of genetic explanations pleaded for lesser punishments for violent behavior of mentally disordered persons. However, there was support for predictions based on the concept of genetic essentialism. People who assume genetic causes of schizophrenia believe in a greater seriousness, tenacity, and pervasiveness of the deviance and hold more social distance against the siblings of mentally disordered persons.

A systematic review of population-based studies found that biogenetic beliefs about the cause of schizophrenia or depression were associated with greater social distance and thus stronger stigmatizing attitudes (1).

Based on the aforementioned and further studies on stigmatization, we have hypothesized that several factors influence whether a given biological model of a given psychiatric disorder will increase stigmatization: (1) disease-specific factors and (2) model-specific factors (10).

(1) Disease-specific factors: biological explanations increase the stigmatization of a given psychiatric disorder, as soon as people think that this disorder is associated with (a) high dangerousness/unpredictability, (b) high psychosocial disability, (c) poor treatment success, and (d) high responsibility for the onset and/or offset of the disease. Among these factors, the most important one is the perceived dangerousness/unpredictability, because this attribution leads people to seek social distance (2).
(2) Model-specific factors: there are different models of psychiatric disorders are, e.g., psychosocial models, the genetic model, the neurotransmitter disturbance model, or the mild encephalitis hypothesis. Model-specific factors can modulate the effects of disease-specific factors in various ways. Model-specific factors can influence the stigmatization, for example, the factor dangerousness/unpredictability either by changing the real dangerousness of people with this disorder or by changing the people’s perception of the dangerousness. The first effect could take place if the model implied an effective treatment against psychosis and/or aggressiveness, the latter if the model convinced people that the disorder was not necessarily associated with dangerousness.

The differential effects of the model-specific factors might be contradictory. For example, genetic explanations of schizophrenia decrease the onset responsibility, but might squash hopes for successful treatments, at least in the laymen’s perception.

Indeed, empirical research on the effects of different models on stigmatization has brought inconsistent results.

According to Rüsch et al. (12), the endorsement of genetic explanations was correlated with a stronger desire for social distance, whereas the endorsement of neurobiological explanations was not correlated with stigmatizing attitudes. In both cases, the attribution of responsibility was reduced.

According to Angermeyer et al. (14), the endorsement of a brain disease hypothesis is associated with increased anger and fear, which is associated with increased social distance. On the contrary, there was no significant association between the endorsement of hereditary factors and social distance, assumedly because the endorsement of hereditary factors increases on the one hand fear and on the other hand prosocial feelings.

In general, biological explanations of schizophrenia increase stigmatization, because schizophrenia has high degrees for three disease-specific factors (dangerousness/unpredictability, psychosocial disability, and poor treatment success). However, it remains an open question whether and in how far neurobiological explanations have a different effect on stigmatization as compared to genetic explanations. This situation is not only due to the inconsistent study results but also due to the rather crude biological explanations used in the studies.

## Anti-Stigma Messages

Accompanying research on stigmatization can contribute to a responsible psychiatric research that will not harm psychiatric patients by involuntarily increasing stigma. Empirical research on stigmatization of mental disorders is particularly necessary for communicating research results to the media and for designing anti-stigma campaigns which are not only well-intended but indeed beneficial for the concerned people. Since stigmatization is a multi-faceted phenomenon, interventions aiming at reducing stigma often have contradictory and unexpected effects.

According to a consensus paper on campaigns to reduce mental health-related stigma, the following message types should be used: (1) recovery-oriented, (2) “see the person,” (3) social inclusion/human rights, and (4) high prevalence of mental disorders (15). Additionally, information on the continuous nature of psychopathological phenomena is recommended for anti-stigma messages (16).

## Influence of the Mild Encephalitis Hypothesis on Stigmatization

We expect that the mild encephalitis hypothesis will have different effects on the stigmatization of schizophrenia.

This hypothesis offers concrete hope for effective therapies with anti-inflammatory drugs for a subgroup of patients diagnosed with schizophrenia (17). Patients will probably accept these drugs better, so that their compliance will improve and the relapse rates might be reduced. With effective and potent drugs, many patients could be treated successfully, so that the dangerousness due to psychosis would vanish. Furthermore, their cognitive decline could be stopped, so that the level of cognitive functioning would be better. Diminished dangerousness and better cognitive functioning will positively affect on their social inclusion.

Because the mild encephalitis hypothesis contains no genetic determinism, but the concept of a genetic vulnerability, we expect that it will reduce the stigmatization of genetic relatives.

The mild encephalitis hypothesis might reduce the stigmatization further because it emphasizes the influence of infections and autoimmune disorders which can principally hit everyone, not only those with a special genetic makeup.

The mild encephalitis hypothesis might not influence the attribution of onset responsibility, because the patients are not responsible for any of the known causes of mild encephalitis. However, the attribution of offset responsibility might change significantly: if effective treatments without severe side effects were available, then the acceptance of the concept “liberty of illness” might diminish. People who refuse effective treatments will be considered as responsible for their enduring mental illness.

Finally, we expect that the stigmatization would be reduced significantly because the mild encephalitis hypothesis would support to shift the organizational authority over patients with schizophrenia from psychiatry to multi-disciplinary institutions combining psychiatry and neurology.

Therefore, we expect that the mild encephalitis hypothesis will contribute to a destigmatization of schizophrenia, of course particularly, if it will lead to effective drug therapies.

## Author Contributions

SM and RR have both contributed to the article with regard to development of ideas. SM wrote the first draft of the manuscript and developed the structure of the paper. Both authors read and approved the final manuscript.

## Conflict of Interest Statement

The authors declare that the research was conducted in the absence of any commercial or financial relationships that could be construed as a potential conflict of interest.
